# A cost comparison of open versus percutaneous approaches to management of large staghorn calculi

**DOI:** 10.4103/0970-1591.38600

**Published:** 2008

**Authors:** Rob Pickard

**Affiliations:** Newcastle University, Newcastle upon Tyne, UK. E-mail: r.s.pickard@ncl.ac.uk

You (should) get what you pay for!

The economics of health care is a major issue worldwide and the evidence base behind decision-making in provision of treatments is increasing. The ‘best’ treatment option for a particular condition in terms of health economics is the one that achieves the desired outcome at the lowest cost-in other words the optimum balance between monetary cost and treatment efficacy. Effectiveness in achieving a set outcome is generally measured in head-to-head comparative studies; typically using a randomized trial design. Comparative costs, as illustrated by the present paper, are often more difficult to pin down. Calculation of precise procedure costs within an institution is problematic since many different treatments will be on offer, patient populations will vary with time, financial responsibility for follow-up may lie elsewhere and many costs are governed by volume of usage.

Cost comparison between centers is even more troublesome since the proportion of the total expenditure accounted for by labour or consumable costs will vary markedly. What the present paper is able to state is that within this single institution, the local combination of patient factors, surgical skill sets, purchasing power and care pathways makes open pyelolithotomy a cheaper option than percutaneous nephrolithotomy (PCNL). Given the non-randomized design and relatively short follow-up period, effectiveness cannot be truly commented upon although the stone-free rate for open surgery is impressive. The generalisability of this finding is more contentious. Concerning effectiveness, we do have evidence from a randomized clinical trial (RCT) that PCNL gives equivalent stone-free rates to open pyelolithotomy with the advantages of less morbidity, shorter hospital stay and less collateral renal damage.[1] In addition even multiple PCNL procedures remain attractive to both patients and health care providers given the ‘keyhole’ nature of access and reduced hospital stay respectively.[2] It is however interesting, that laparoscopic pyelolithotomy is currently being attempted, which may suggest that some still feel that direct access and removal of the intact stone is preferable.^3^ At the very least, this paper should encourage all units, however they are financed, to audit outcomes and estimate costs in order to ensure that they are offering the ‘best’ that is locally available to their customers.

## References

[1] Al-Kohlany KM, Shokeir AA, Mosbah A, Mohsen T, Shoma AM, Eraky I (2005). Treatment of complete staghorn stones: A prospective randomized comparison of open surgery versus percutaneous nephrolithotomy. J Urol.

[2] Ansari MS, Gupta NP (2003). Impact of socioeconomic status in etiology and management of urinary stone disease. Urol Int.

[3] Nambirajan T, Jeschke S, Albqami N, Abukora F, Leeb K, Janetschek G (2005). Role of laparoscopy in management of renal stones: Single-center experience and review of literature. J Endourol.

